# Supplementary material for: Single Crystal FLIM Characterization of Clofazimine Loaded in Silica-Based Mesoporous Materials and Zeolites
Source: Int J Mol Sci. 2019 Jun 12;20(12):2859. doi: 10.3390/ijms20122859 (PMC6627708; doi:10.3390/ijms20122859)
Supplement: Supplementary file 1 [file ijms-20-02859-s001.pdf]

International Journal of
Molecular Sciences

MDPI

Article

Single crystal FLIM characterization of clofazimine loaded in silica-based mesoporous materials and zeolites

Lorenzo Angiolini [1], Boiko Cohen [1,*] and Abderrazzak Douhal [1,*]

[1] Departamento de Química Física, Facultad de Ciencias Ambientales y Bioquímica, and INAMOL, Universidad de Castilla-La Mancha, Avenida Carlos III, S/N, 45071 Toledo, Spain; Lorenzo.Angiolini@uclm.es

* Correspondence: Boyko.Koen@uclm.es (B.C.); abderrazzak.douhal@uclm.es (A.D.); Tel.: +34-925-268800 (ext. 5571) (B.C.); +34-925-265717 (A.D.)

Received: 20 April 2019; Accepted: 10 June 2019; Published: 12 June 2019

Supplementary material

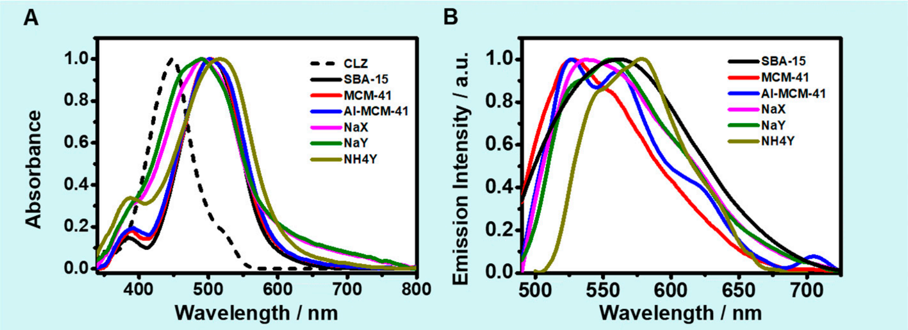

Figure S1. (**A**) Absorption and (**B**) emission spectra of CLZ in a DCM solution and interacting with the indicated silica materials (4.3 × 10^{-5} M) in DCM suspensions. The excitation wavelength for the emission spectra was set at 470 nm.

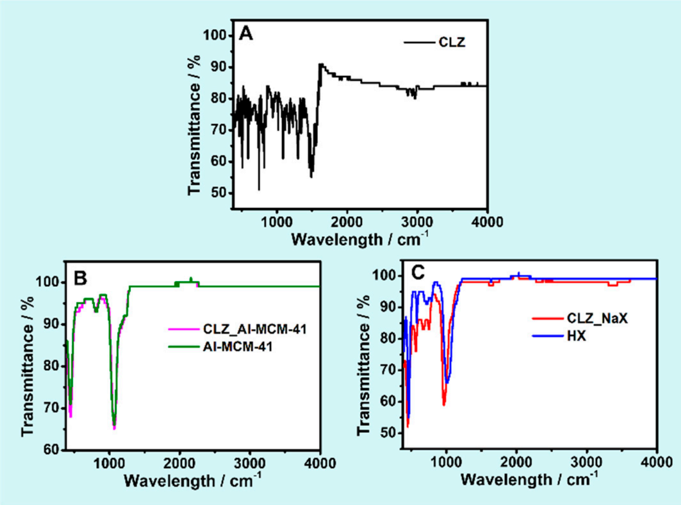

Figure S2. FTIR spectra of (**A**) CLZ alone, (**B**) CLZ loaded in Al-MCM-41 and the empty Al-MCM-41supporta and (**C**) CLZ loaded in NaX and empty HY zeolite.

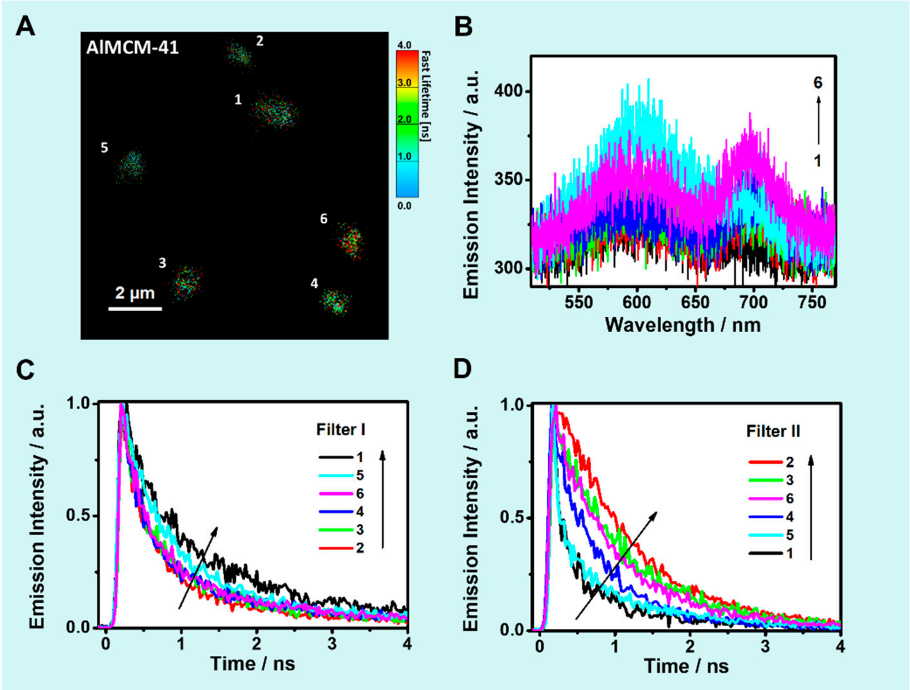

Figure S3. (**A**) FLIM image, (**B**) emission spectra, and lifetime decays normalized to the maximum of intensity and collected using (**C**) a 510-570 nm bandpass filter I and (**D**) a 700 nm longpass filter II upon excitation at 470 nm of CLZ@Al-MCM-41 (4.3 × 10^{-5} M). The labelling in (B)-(D) corresponds to that in (A).

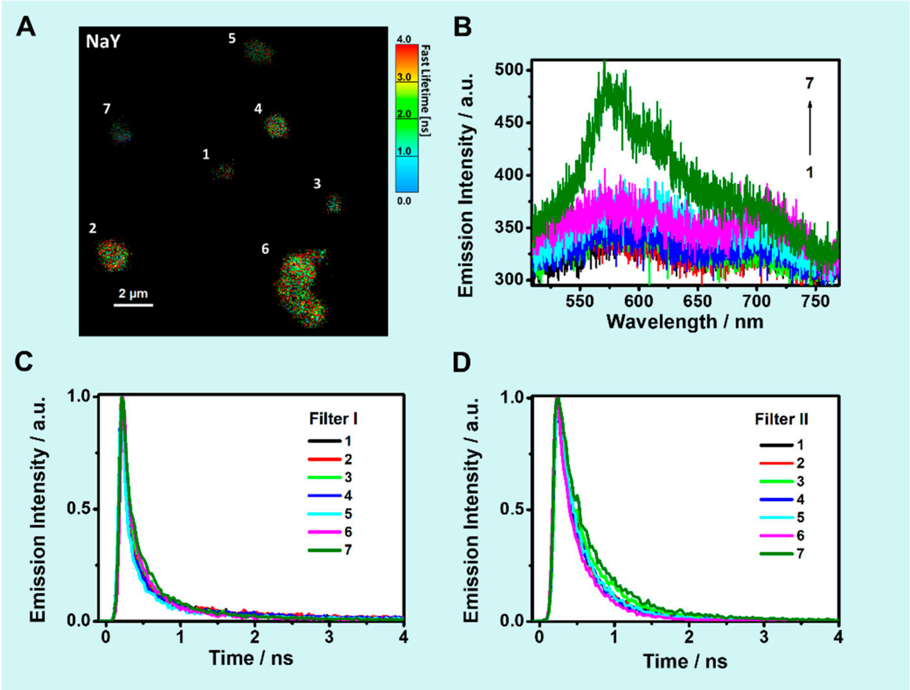

Figure S4. (**A**) FLIM image, (**B**) emission spectra, and lifetime decays normalized to the maximum of intensity and collected using (**C**) a 510-570 nm bandpass filter I and (**D**) a 700 nm longpass filter II upon excitation at 470 nm of CLZ@NaY (4.3 × 10^{-5} M). The labelling in (B)-(D) corresponds to that in (A).

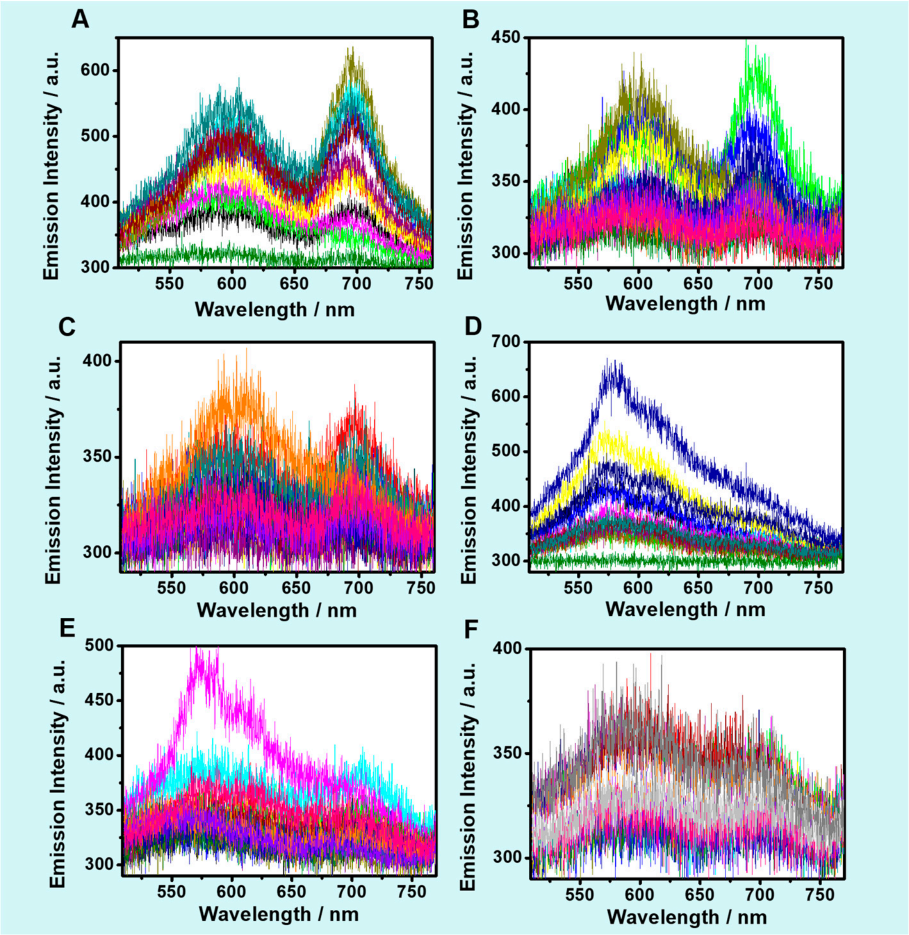

Figure S5. Single crystals emission spectra of (**A**) CLZ@SBA-15; (**B**) CLZ@MCM-41; (**C**) CLZ@Al-MCM-41; (**D**) CLZ@NaX; (**E**) CLZ@NaY; (**F**) CLZ@NH4Y (4.3 × 10^{-5} M) observed upon excitation at 470 nm.

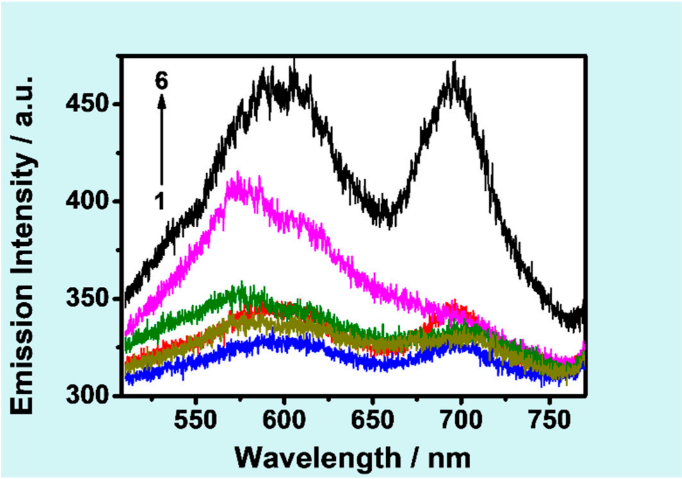

Figure S6. Averaged emission spectra upon excitation at 470 nm of CLZ-loaded single crystals (1→6: Al-MCM-41, NH4Y, MCM-41, NaY, NaX, SBA-15; 4.3 × 10^{-5} M).

Table S1. Ratio (I_{700}/I_{600}) of CLZ@SBA-15 (4.3×10^{-5} M) emission intensity maxima collected at 600 nm and 700 nm upon excitation at 470 nm.

Particle	I_{700}/I_{600}	Average
F1 1 (1)	0.50	
F1 2	0.59	
F1 3	0.85	
F2 1	1.70	
F2 2 (3)	0.76	
F2 3	0.74	
F3 1 (4)	1.12	0.92
F3 2 (6)	1.02	
F4 1 (2)	1.07	
F4 2	0.74	
F4 3	0.35	
F4 4 (5)	1.28	
F4 5 (7)	1.28	

Table S2. Ratio (I_{700}/I_{600}) of CLZ@MCM-41 (4.3×10^{-5} M) emission intensity maxima collected at 600 nm and 700 nm upon excitation at 470 nm.

Particle	I_{700}/I_{600}	Average
F1 1	0.61	
F1 2 (4)	1.14	
F1 3 (3)	0.40	
F1 4	1.34	
F1 5 (5)	0.35	
F1 6	1.06	
F2 1	1.19	
F2 2 (2)	1.39	
F2 3	1.55	1.08
F2 4	1.62	
F2 5	2.14	
F2 6	1.10	
F3 1 (1)	0.54	
F3 2	0.67	
F3 3 (6)	1.79	
F3 4	0.88	
F3 5	0.53	

Table S3. Ratio (I_{700}/I_{600}) of CLZ@Al-MCM-41 (4.3×10^{-5} M) emission intensity maxima collected at 600 nm and 700 nm upon excitation at 470 nm.

Particle	I_{700}/I_{600}	Average
F1 1 (2)	1.16	
F1 2	0.73	
F1 3	1.24	
F1 4	1.11	
F1 5	0.80	
F1 6	0.95	
F2 1	0.47	
F2 2	0.62	
F2 3	0.95	0.98
F2 4 (1)	0.40	
F2 5 (5)	0.39	
F3 1 (3)	1.00	
F3 2 (6)	1.35	
F3 3	1.64	
F3 4 (4)	1.25	
F3 5	1.29	
F3 6	1.29	

Table S4. Ratio (I_{700}/I_{570}) of CLZ@NaX (4.3×10^{-5} M) emission intensity maxima collected at 570 nm and 700 nm upon excitation at 470 nm.

Particle	I_{700}/I_{570}	Average
F1 1 (4)	0.25	0.33
F1 2	0.44	
F1 3 (2)	0.30	
F2 1	0.67	
F2 2 (5)	0.23	
F2 3 (3)	0.31	
F2 4 (7)	0.36	
F3 1	0.28	
F3 2 (1)	0.20	
F3 3	0.26	
F3 4 (6)	0.34	
F4 1	0.36	
F4 2	0.28	
F4 3	0.31	

Table S5. Ratio (I_{700}/I_{570}) of CLZ@NaY (4.3×10^{-5} M) emission intensity maxima collected at 570 nm and 700 nm upon excitation at 470 nm.

Particle	I_{700}/I_{570}	Average
F1 1 (5)	0.55	
F1 2	0.29	
F1 3 (7)	0.28	
F2 1 (4)	0.60	
F2 2	0.33	
F2 3 (1)	0.76	
F2 4 (3)	0.39	
F2 5	0.55	
F3 1	0.38	0.61
F3 2	0.60	
F3 3	1.31	
F3 4	1.20	
F3 5 (6)	0.91	
F4 1	0.62	
F4 2	0.30	
F4 3	0.81	
F4 4 (2)	0.43	

Table S6. Ratio (I_{700}/I_{570}) of CLZ@NH4Y (4.3 × 10^{-5} M) emission intensity maxima collected at 570 nm and 700 nm upon excitation at 470 nm.

Particle	I_{700}/I_{570}	Average
F1 1 (7)	0.56	
F1 2 (6)	0.52	
F1 3	1.46	
F1 4	1.16	
F1 5	0.85	
F1 6 (1)	0.83	
F2 1	0.93	
F2 2	1.38	
F2 3 (5)	0.73	
F2 4 (4)	0.40	0.84
F2 5	0.75	
F2 6 (3)	0.85	
F2 7	1.06	
F3 1	0.78	
F3 2	0.54	
F3 3 (2)	0.92	
F3 4	0.65	
F3 5	0.78	
F3 6	0.88	

Table S7. Deconvolution results of the averaged emission spectra of the indicated CLZ@silica composites (4.3 × 10^{-5} M) collected upon excitation at 470 nm.

Material	Peak	Position (nm)	FWHM (cm^{-1})	Amplitude (%)
SBA-15	1	535	1483	7
	2	572	1143	6
	3	605	2047	51
	4	696	1026	36
MCM-41	1	532	1418	7
	2	572	1452	10
	3	605	2003	50
	4	697	911	33
Al-MCM-41	1	539	1041	4
	2	572	1060	9
	3	605	1916	54
	4	696	941	33
NaX	1	535	1746	16
	2	572	1082	16
	3	605	2324	54
	4	699	1417	14
NaY	1	535	2007	21
	2	572	1026	8
	3	605	2343	48
	4	701	1262	23
NH4Y	1	535	1782	6
	2	572	999	7
	3	605	2651	61
	4	700	1228	27

Table S8. Values of time constants (τ_i), normalized (to 100) pre-exponential factors (a_i), fractional contributions ($c_i = \tau_i a_i / \Sigma\tau_i a_i$) and standard deviations (σi) of the multiexponential function used to fit the fluorescence lifetime decays of CLZ@MCM-41 composites (4.3×10^{-5} M) collected using the indicated filters, upon excitation at 470 nm.

Filter (nm)	Sample	τ_1 (ns)[1]	a_1 (%)	c_1 (%)	τ_2 (ns)[2]	a_2 (%)	c_2 (%)	τ_3 (ns)[2]	a_3 (%)	c_3 (%)
510-570	F1 1	0.1	52	10	0.7	42 (87)	54 (60)	3.2	6 (13)	36 (40)
	F1 2 (4)	0.1	44	8	0.6	48 (86)	54 (58)	2.7	8 (14)	38 (42)
	F1 3 (3)	0.1	40	6	0.7	52 (86)	58 (62)	2.7	8 (14)	36 (38)
	F1 4	0.1	55	9	0.8	38 (84)	48 (53)	3.7	7 (16)	43 (47)
	F1 5 (5)	-	-	-	0.7	81	53	2.6	19	47
	F1 6	-	-	-	0.6	84	53	2.7	16	47
	F2 1	0.1	46	9	0.6	48 (88)	55 (60)	2.9	6 (12)	36 (40)
	F2 2 (2)	0.1	7	1	0.7	77 (82)	50 (50)	3.2	16 (18)	49 (50)
	F2 3	0.1	52	7	0.9	41 (85)	47 (51)	4.8	7 (15)	46 (49)
	F2 4	0.1	50	8	0.6	39 (77)	37 (40)	2.9	11 (23)	55 (60)
	F2 5	0.1	33	4	0.8	54 (81)	50 (52)	3.1	13 (19)	46 (48)
	F2 6	-	-	-	0.8	76	41	3.6	24	59
	F3 1 (1)	0.1	52	9	0.7	41 (86)	49 (53)	3.6	7 (14)	42 (47)
	F3 2	0.1	52	8	0.7	40 (83)	44 (48)	3.8	8 (17)	48 (52)
	F3 3 (6)	0.1	46	8	0.7	47 (87)	55 (60)	3.1	7 (13)	37 (40)
	F3 4	-	-	-	0.6	81	50	2.6	19	50
	F3 5	0.1	53	9	0.7	40 (84)	47 (52)	3.4	7 (16)	44 (48)
700	F1 1	0.1	39	6	0.8	43 (71)	49 (52)	1.8	18 (29)	45 (48)
	F1 2 (4)	-	-	-	0.8	61	43	1.7	39	57
	F1 3 (3)	0.1	64	14	0.9	29 (82)	55 (64)	2.2	7(18)	31 (36)
	F1 4	-	-	-	1	78	62	2.2	22	38
	F1 5 (5)	0.1	69	14	0.9	21 (67)	40 (46)	2.1	10 (33)	46 (53)
	F1 6	-	-	-	1	55	39	1.9	45	61
	F2 1	-	-	-	0.7	69	49	1.6	31	51
	F2 2 (2)	-	-	-	0.8	72	51	1.9	28	49
	F2 3	-	-	-	1.1	80	67	2.2	20	33
	F2 4	-	-	-	1	87	75	2.3	13	25
	F2 5	-	-	-	1.3	85	75	2.4	15	25
	F2 6	-	-	-	1.3	84	71	2.8	16	29
	F3 1 (1)	0.1	28	4	0.7	55 (76)	53 (55)	1.8	17 (24)	43 (45)
	F3 2	0.1	33	4	0.9	55 (81)	62 (65)	2.1	12 (19)	34 (35)
	F3 3 (6)	-	-	-	1.0	81	66	2.1	19	34
	F3 4	0.1	21	2	1.2	61 (78)	64 (65)	2.2	18 (22)	34 (35)
	F3 5	0.1	51	8	0.7	42 (84)	48 (52)	3.4	7 (16)	44 (48)

[1] τ_1 was fixed in the fit; [2] The error in lifetimes values is <10%.

Table S8. Values of time constants (τ_i), normalized (to 100) pre-exponential factors (a_i), fractional contributions ($c_i = \tau_i a_i / \Sigma\tau_i a_i$) and standard deviations (σi) of the multiexponential function used to fit the fluorescence lifetime decays of CLZ@Al-MCM-41 composites (4.3×10^{-5} M) collected using the indicated filters, upon excitation at 470 nm.

Filter (nm)	Sample	τ_1 (ns)[1]	a_1 (%)	c_1 (%)	τ_2 (ns)[2]	a_2 (%)	c_2 (%)	τ_3 (ns)[2]	a_3 (%)	c_3 (%)
510-570	F1 1 **(2)**	0.1	51	10	0.6	42 (87)	50 (56)	3.1	7 (13)	40 (44)
	F1 2	-	-	-	0.5	88	57	2.8	12	43
	F1 3	0.1	70	24	0.5	28 (93)	48 (64)	4	2 (7)	28 (36)
	F1 4	-	-	-	0.7	87	59	3.2	13	41
	F1 5	-	-	-	0.6	88	55	3.5	12	45
	F1 6	0.1	59	10	0.8	35 (85)	46 (52)	4.2	6 (15)	44 (48)
	F2 1	0.1	42	5	0.8	45 (78)	46 (48)	3	13 (22)	49 (52)
	F2 2	0.1	46	7	0.7	46 (86)	50 (54)	3.5	8 (14)	43 (46)
	F2 3	0.1	73	25	0.5	24 (90)	43 (57)	3.4	3 (10)	32 (43)
	F2 4 **(1)**	0.1	41	5	0.9	48 (82)	49 (51)	3.9	11 (18)	46 (49)
	F2 5 **(5)**	0.1	47	7	0.8	44 (84)	53 (57)	3.2	8 (16)	40 (43)
	F3 1 **(3)**	0.1	54	10	0.7	40 (86)	51 (56)	3.2	7 (14)	39 (44)
	F3 2 **(6)**	0.1	58	10	0.7	36 (84)	44 (49)	3.9	7 (16)	46 (51)
	F3 3	0.1	64	14	0.6	30 (83)	39 (45)	3.5	6 (17)	47 (55)
	F3 4 **(4)**	0.1	61	12	0.7	32 (83)	44 (49)	3.6	4 (17)	44 (51)
	F3 5	0.1	69	22	0.5	27 (88)	44 (56)	2.8	4 (12)	34 (44)
700	F1 1 **(2)**	-	-	-	1.0	98	94	3.0	2	6
	F1 2	-	-	-	0.7	86	72	1.7	14	28
	F1 3	-	-	-	0.6	79	63	1.3	21	37
	F1 4	0.1	27	4	0.8	58 (79)	60 (63)	1.8	15 (21)	36 (37)
	F1 5	-	-	-	0.8	79	60	2	21	40
	F1 6	-	-	-	0.8	87	73	1.9	13	27
	F2 1	0.1	48	7	1.1	49 (95)	83 (89)	4	3 (5)	10 (11)
	F2 2	0.1	43	6	1	51 (90)	72 (77)	2.6	6 (10)	22 (23)
	F2 3	-	-	-	0.5	82	65	1.2	18	35
	F2 4 **(1)**	0.1	77	27	0.6	17 (76)	36 (50)	1.9	6 (24)	37 (50)
	F2 5 **(5)**	0.1	71	18	0.8	22 (74)	44 (54)	2.0	7 (26)	38 (46)
	F3 1 **(3)**	0.1	22	3	0.9	68 (88)	74 (76)	2.0	10 (12)	23 (24)
	F3 2 **(6)**	0.1	25	3	0.8	63 (83)	68 (70)	1.7	12 (17)	29 (30)
	F3 3	-	-	-	0.6	88	65	1.5	12	35
	F3 4 **(4)**	0.1	30	6	0.6	62 (88)	69 (74)	1.6	8 (12)	25 (26)
	F3 5	0.1	23	5	0.5	64 (83)	62 (65)	1.3	13 (17)	33 (35)

[1] τ_1 was fixed in the fit; [2] The error in lifetimes values is <10%.

Table S10. Values of time constants (τ_i), normalized (to 100) pre-exponential factors (a_i), fractional contributions ($c_i = \tau_i a_i / \Sigma\tau_i a_i$) and standard deviations (σi) of the multiexponential function used to fit the fluorescence lifetime decays of CLZ@SBA-15 composites (4.3 × 10^{-5} M) collected using the indicated filters, upon excitation at 470 nm.

Filter (nm)	Sample	τ_1 (ns)[1]	a_1 (%)	c_1 (%)	τ_2 (ns)[2]	a_2 (%)	c_2 (%)	τ_3 (ns)[2]	a_3 (%)	c_3 (%)
510-570	F1 1 **(1)**	0.1	43	8	0.6	48 (84)	54 (59)	2.2	9 (16)	38 (41)
	F1 2	-	-	-	0.5	82	52	2.2	18	48
	F1 3	0.1	36	5	0.8	54 (83)	54 (57)	3	10 (17)	41 (43)
	F2 1	0.1	50	11	0.6	45 (91)	61 (69)	2.8	5 (9)	28 (31)
	F2 2 **(3)**	0.1	49	11	0.6	47 (91)	61 (69)	2.9	4 (9)	28 (31)
	F2 3	0.1	41	9	0.5	52 (89)	58 (64)	2.2	7 (11)	33 (36)
	F3 1 **(4)**	0.1	53	11	0.6	42 (89)	55 (62)	3.0	5 (11)	34 (38)
	F3 2 **(6)**	0.1	44	9	0.6	51 (91)	64 (71)	2.6	5 (9)	27 (29)
	F4 1 **(2)**	0.1	50	12	0.5	45 (90)	56 (64)	2.5	5(10)	32 (36)
	F4 2	0.1	39	6	0.7	51 (85)	56 (59)	2.6	10 (15)	38 (41)
	F4 3	0.1	39	7	0.6	54 (89)	62 (67)	2.3	7 (11)	31 (33)
	F4 4 **(5)**	0.1	58	16	0.5	38 (91)	52 (62)	2.9	4 (9)	32 (38)
	F4 5 **(7)**	0.1	37	7	0.6	56 (89)	64 (69)	2.1	7 (11)	29 (31)
700	F1 1 **(1)**	0.1	46	7	0.9	42 (78)	57 (61)	2.0	12 (22)	36 (39)
	F1 2	0.1	25	3	0.8	52 (69)	47 (49)	1.9	23 (31)	50 (51)
	F1 3	-	-	-	1	72	57	2	28	43
	F2 1	-	-	-	0.9	80	68	1.7	20	32
	F2 2 **(3)**	-	-	-	0.9	80	68	1.7	20	32
	F2 3	0.1	37	6	0.8	49 (78)	59 (63)	1.7	14 (22)	35 (37)
	F3 1 **(4)**	0.1	23	3	0.8	60 (79)	60 (62)	1.7	17 (21)	37 (38)
	F3 2 **(6)**	-	-	-	0.9	71	58	1.6	29	42
	F4 1 **(2)**	0.1	21	2	0.8	58 (73)	55 (56)	1.7	21 (27)	43 (44)
	F4 2	0.1	44	6	1	42 (76)	57 (61)	2	14 (24)	37 (39)
	F4 3	0.1	81	32	0.6	14 (74)	34 (50)	1.7	5 (26)	34 (50)
	F4 4 **(5)**	-	-	-	0.7	70	52	1.5	30	48
	F4 5 **(7)**	-	-	-	1	71	58	1.8	29	42

[1] τ_1 was fixed in the fit; [2] The error in lifetimes values is <10%.

Table S11. Values of time constants (τ_i), normalized (to 100) pre-exponential factors (a_i), fractional contributions ($c_i = \tau_i a_i / \Sigma\tau_i a_i$) and standard deviations (σi) of the multiexponential function used to fit the fluorescence lifetime decays of CLZ@NaX composites (4.3×10^{-5} M) collected using the indicated filters, upon excitation at 470 nm.

Filter (nm)	Sample	τ_1 (ns)[1]	a_1 (%)	c_1 (%)	τ_2 (ns)[2]	a_2 (%)	c_2 (%)	τ_3 (ns)[2]	a_3 (%)	c_3 (%)
510-570	F1 1 **(4)**	0.1	62	17	0.6	34 (91)	55 (66)	3.0	4 (9)	28 (34)
	F1 2	0.1	71	20	0.6	26 (88)	45 (56)	3.5	3 (12)	35 (44)
	F1 3 **(2)**	0.1	63	17	0.6	33 (90)	53 (64)	3.0	4 (10)	30 (36)
	F2 1	0.1	52	10	0.7	40 (83)	50 (56)	2.7	8 (17)	40 (44)
	F2 2 **(5)**	0.1	65	20	0.5	32 (91)	49 (61)	3.2	3 (9)	31 (39)
	F2 3 **(3)**	0.1	60	16	0.6	37 (91)	57 (68)	2.9	3 (9)	27 (32)
	F2 4 **(7)**	0.1	64	18	0.6	32(91)	54 (66)	3.1	3 (9)	28 (34)
	F3 1	0.1	62	19	0.5	34 (89)	51 (63)	2.4	4 (11)	30 (37)
	F3 2 **(1)**	0.1	61	14	0.6	34 (86)	48 (56)	2.9	5 (14)	37 (44)
	F3 3	0.1	62	15	0.7	35 (91)	57 (67)	3.7	3 (9)	28 (33)
	F3 4 **(6)**	0.1	61	13	0.7	34 (87)	51 (59)	3.3	5 (13)	36 (41)
	F4 1	0.1	66	16	0.7	30 (87)	51 (61)	3	4 (13)	33 (39)
	F4 2	0.1	67	16	0.7	29 (88)	48 (57)	3.9	4 (12)	36 (43)
	F4 3	0.1	60	14	0.7	36 (90)	58 (68)	3.1	4 (10)	28 (32)
700	F1 1 **(4)**	-	-	-	0.4	87	67	1.3	13	33
	F1 2	0.1	58	23	0.4	38 (92)	59 (77)	1.3	4 (8)	18 (23)
	F1 3 **(2)**	0.1	43	11	0.5	53 (92)	68 (76)	1.8	5 (8)	21 (24)
	F2 1	0.1	43	11	0.5	51 (89)	65 (73)	1.5	6 (11)	24 (27)
	F2 2 **(5)**	0.1	44	12	0.5	52 (94)	70 (80)	1.9	4 (6)	18 (20)
	F2 3 **(3)**	0.1	39	9	0.5	55 (90)	67 (74)	1.6	6 (10)	24 (26)
	F2 4 **(7)**	0.1	47	14	0.5	48 (92)	70 (81)	1.6	4 (8)	16 (19)
	F3 1	0.1	41	10	0.5	52 (88)	64 (71)	1.5	7 (12)	26 (29)
	F3 2 **(1)**	0.1	48	13	0.5	48 (92)	66 (76)	1.8	4 (8)	21 (24)
	F3 3	0.1	52	16	0.5	44 (93)	66 (79)	1.7	4 (7)	18 (21)
	F3 4 **(6)**	0.1	61	21	0.5	36 (93)	61 (77)	2.1	3 (7)	18 (23)
	F4 1	0.1	48	14	0.5	47 (91)	66 (77)	1.5	5 (9)	20 (23)
	F4 2	0.1	57	20	0.4	38 (89)	56 (70)	1.4	5 (11)	24 (30)
	F4 3	0.1	60	20	0.5	37 (92)	62 (77)	1.8	3 (8)	18 (23)

[1] τ_1 was fixed in the fit; [2] The error in lifetimes values is <10%.

Table S12. Values of time constants (τ_i), normalized (to 100) pre-exponential factors (a_i), fractional contributions ($c_i = \tau_i a_i / \Sigma \tau_i a_i$) and standard deviations (σi) of the multiexponential function used to fit the fluorescence lifetime decays of CLZ@NaY composites (4.3×10^{-5} M) collected using the indicated filters, upon excitation at 470 nm.

Filter (nm)	Sample	τ_1 (ns)[1]	a_1 (%)	c_1 (%)	τ_2 (ns)[2]	a_2 (%)	c_2 (%)	τ_3 (ns)[2]	a_3 (%)	c_3 (%)
510-570	F1 1 (5)	0.1	88	49	0.5	11 (92)	30 (58)	4.1	1 (8)	21 (42)
	F1 3 (7)	0.1	74	31	0.5	24 (93)	51 (74)	2.4	2 (7)	18 (26)
	F2 1 (4)	0.1	88	49	0.4	11 (90)	25 (50)	3.8	1 (10)	26 (50)
	F2 2	0.1	60	14	0.6	36 (89)	51 (59)	3.3	4 (11)	35 (41)
	F2 3 (1)	0.1	85	40	0.6	13 (89)	35 (59)	3.5	2 (11)	25 (41)
	F2 4 (3)	0.1	81	38	0.5	16 (88)	37 (59)	2.4	3 (12)	25 (41)
	F2 5	0.1	86	32	0.8	11 (85)	34 (50)	4.7	2 (15)	34 (50)
	F3 1	0.1	86	45	0.5	13 (93)	34 (62)	4	1 (7)	21 (38)
	F3 2	0.1	87	54	0.4	12 (95)	30 (65)	4	1 (5)	16 (35)
	F3 3	0.1	84	42	0.5	15 (93)	38 (65)	3.7	1 (7)	20 (35)
	F3 4	0.1	84	46	0.4	15 (94)	33 (60)	4	1 (6)	21 (40)
	F3 5 (6)	0.1	78	39	0.4	21 (94)	42 (68)	2.8	1 (6)	19 (32)
	F4 1	0.1	83	36	0.5	15 (90)	33 (52)	4.1	2 (10)	31 (48)
	F4 3	0.1	87	33	0.7	11 (85)	30 (45)	4.7	2 (15)	37 (55)
	F4 4 (2)	0.1	88	36	0.7	10 (86)	30 (47)	4.8	2 (14)	34 (53)
700	F1 1 (5)	0.1	41	13	0.4	56 (95)	75 (87)	1.2	3 (5)	12 (13)
	F1 3 (7)	-	-	-	0.4	90	74	1.3	10	26
	F2 1 (4)	0.1	43	6	0.4	55 (96)	91 (96)	1.2	2 (4)	3 (4)
	F2 2	0.1	25	5	0.5	69 (91)	74 (78)	1.5	6 (9)	21 (22)
	F2 3 (1)	0.1	42	14	0.4	54 (93)	73 (85)	1.0	4 (7)	13 (15)
	F2 4 (3)	0.1	42	14	0.4	55 (95)	73 (85)	1.3	3 (5)	13 (15)
	F2 5	0.1	54	22	0.4	45 (98)	73 (94)	1.7	1 (2)	5 (6)
	F3 1	0.1	45	16	0.4	53 (97)	75 (89)	1.5	2 (3)	9 (11)
	F3 2	0.1	52	21	0.4	47 (98)	74 (93)	1.3	1 (2)	5 (7)
	F3 3	0.1	85	44	0.5	14 (93)	36 (65)	3.7	1 (2)	20 (35)
	F3 4	0.1	38	12	0.4	59 (96)	77 (88)	1.2	3 (4)	11 (12)
	F3 5 (6)	0.1	44	15	0.4	53 (94)	71 (83)	1.3	3 (6)	14 (17)
	F4 1	0.1	38	12	0.4	58 (94)	73 (83)	1.4	4 (6)	15 (17)
	F4 3	0.1	53	22	0.4	46 (99)	74 (95)	1.7	1 (1)	4 (5)
	F4 4 (2)	0.1	51	20	0.4	48 (98)	74 (92)	1.6	1 (2)	6 (8)

[1] τ_1 was fixed in the fit; [2] The error in lifetimes values is <10%.

Table S13. Values of time constants (τ_i), normalized (to 100) pre-exponential factors (a_i), fractional contributions ($c_i = \tau_i a_i / \Sigma\tau_i a_i$) and standard deviations (σi) of the multiexponential function used to fit the fluorescence lifetime decays of CLZ@NH4Y composites (4.3×10^{-5} M) collected using the indicated filters, upon excitation at 470 nm.

Filter (nm)	Sample	τ_1 (ns)[1]	a_1 (%)	c_1 (%)	τ_2 (ns)[2]	a_2 (%)	c_2 (%)	τ_3 (ns)[2]	a_3 (%)	c_3 (%)
510-570	F1 1 (7)	0.1	79	45	0.4	20 (96)	46 (84)	2.1	1 (4)	9 (16)
	F1 2 (6)	0.1	76	41	0.4	23 (97)	49 (83)	2.7	1 (3)	10 (17)
	F1 3	0.1	87	57	0.4	12 (98)	33 (77)	4.8	1 (2)	10 (23)
	F1 4	0.1	90	55	0.5	9 (92)	26 (58)	4.1	1 (8)	19 (42)
	F1 5	0.1	83	56	0.3	16 (97)	33 (76)	2.9	1 (3)	11 (24)
	F1 6 (1)	0.1	92	57	0.5	7 (91)	23 (53)	4.7	1 (9)	20 (47)
	F2 1	0.1	87	58	0.4	12 (96)	32 (75)	3.2	1 (4)	10 (25)
	F2 2	0.1	85	56	0.4	14 (96)	36 (83)	1.8	1 (4)	8 (17)
	F2 3 (5)	0.1	88	53	0.4	11 (95)	28 (59)	4.8	1 (5)	19 (41)
	F2 4 (4)	0.1	91	46	0.4	18 (96)	40 (74)	3.2	1 (4)	14 (26)
	F2 5	0.1	84	56	0.4	15 (97)	39 (89)	1.5	1 (3)	5 (11)
	F2 6 (3)	0.1	88	57	0.4	11 (95)	29 (67)	3.9	1 (5)	14 (33)
	F2 7	0.1	93	51	0.7	6 (87)	25 (50)	4.7	1 (13)	24 (50)
	F3 1	0.1	92	50	0.6	7 (86)	23 (46)	4.4	1 (14)	27 (54)
	F3 2	0.1	91	50	0.5	8 (87)	22 (43)	4.5	1 (13)	28 (57)
	F3 3 (2)	0.1	89	58	0.4	10 (94)	27 (65)	3.4	1 (6)	15 (35)
	F3 4	0.1	91	50	0.5	8 (87)	22 (44)	4.2	1 (13)	28 (56)
	F3 5	0.1	84	53	0.4	15 (97)	39 (83)	3.2	1 (3)	8 (17)
	F3 6	0.1	87	43	0.5	12 (91)	30 (53)	4.5	1 (9)	27 (47)
700	F1 1 (7)	0.1	45	15	0.4	52 (95)	73 (86)	1.2	3 (5)	12 (14)
	F1 2 (6)	0.1	46	17	0.4	53 (98)	78 (94)	1.9	1 (2)	5 (6)
	F1 3	0.1	46	17	0.4	53 (98)	79 (94)	1.2	1 (2)	4 (6)
	F1 4	0.1	42	15	0.4	57 (98)	80 (94)	1.3	1 (2)	5 (6)
	F1 5	0.1	38	12	0.4	60 (96)	79 (90)	1.2	2 (4)	9 (10)
	F1 6 (1)	0.1	42	14	0.4	56 (96)	78 (91)	1.1	2 (4)	8 (9)
	F2 1	0.1	43	15	0.4	54 (95)	76 (89)	0.9	3 (5)	9 (11)
	F2 2	0.1	39	13	0.4	58 (96)	77 (89)	1.2	3 (4)	10 (11)
	F2 3 (5)	0.1	43	15	0.4	55 (97)	78 (91)	1.2	2 (3)	7 (9)
	F2 4 (4)	0.1	43	15	0.4	56 (98)	79 (93)	1.5	1 (2)	6 (7)
	F2 5	0.1	46	21	0.3	52 (96)	71 (90)	0.9	2 (4)	8 (10)
	F2 6 (3)	0.1	43	15	0.4	55 (97)	78 (92)	1.1	2 (3)	7 (8)
	F2 7	0.1	60	27	0.4	39 (99)	71 (98)	1	1 (1)	2 (2)
	F3 1	0.1	51	20	0.4	48 (99)	77 (97)	1.8	1 (1)	3 (3)

F3 2	0.1	66	33	0.4	33 (99)	65 (97)	1.1	1 (1)	2 (3)
F3 3 (2)	0.1	44	16	0.4	54 (97)	78 (93)	1.1	2 (3)	6 (7)
F3 4	0.1	45	17	0.4	54 (99)	81 (97)	1.9	1 (1)	2 (3)
F3 5	0.1	43	15	0.4	55 (96)	75 (89)	1.3	2 (4)	10 (11)
F3 6	0.1	54	22	0.4	45 (98)	73 (93)	1.5	1 (2)	5 (7)

[1] τ_1 was fixed in the fit; [2] The error in lifetimes values is <10%.

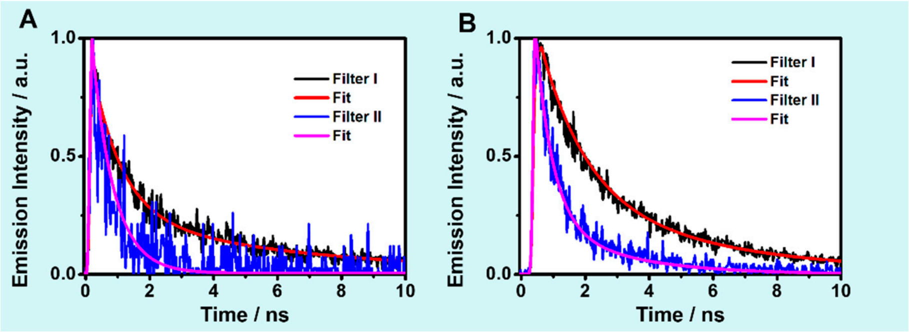

Figure S7. Lifetime decays normalized to the maximum of intensity of (**A**) CLZ@MCM-41 (4.3×10^{-7} M) and (**B**) CLZ@NaX (4.3×10^{-9} M) collected upon excitation at 470 nm.

Table S14. Values of time constants (i) and normalized (to 100) pre-exponential factors (a_i) of the multiexponential function used to fit the fluorescence lifetime decays of CLZ@MCM-41 composites (4.3×10^{-7} M), collected using the indicated filters, upon excitation at 470 nm.

Filter (nm)	τ_2 (ns)[1]	a_2 (%)	τ_3 (ns)[1]	a_3 (%)
510-570	0.5	80	4.2	20
	1.0	73	5.2	27
	0.9	73	6.0	27
	0.8	70	5.0	30
	1.0	72	4.7	28
	1.0	72	5.4	28
	0.9	77	6.0	23
	1.0	75	7.1	25
	0.8	72	5.4	28
	0.9	73	5.6	27
	0.6	87	2.6	13
	0.9	74	5.2	26
	0.8	73	5.2	27
	1.2	78	5.6	22
	0.8	67	4.2	33
700	0.4	86	2.1	14
	0.4	83	3.0	17
	0.9	100	-	-
	0.6	100	-	-
	1.0	100	-	-
	1.0	100	-	-
	0.8	100	-	-
	1.1	100	-	-
	1.0	100	-	-
	1.2	100	-	-
	0.6	82	1.9	18
	0.7	100	-	-
	1.3	100	-	-
	1.0	100	-	-
	0.8	100	-	-

[1] The error in lifetimes values is <10%.

Table S15. Values of time constants ($_i$) and normalized (to 100) pre-exponential factors (a_i) of the multiexponential function used to fit the fluorescence lifetime decays of CLZ@NaX composites (4.3 × 10^{-9} M), collected using the indicated filters, upon excitation at 470 nm.

Filter (nm)	τ_2 (ns)[1]	a_2 (%)	τ_3 (ns)[1]	a_3 (%)
510-570	1.6	58	5.2	42
	0.9	64	4.5	36
	1.2	67	4.5	33
	1.6	79	6.4	21
	1.8	57	5.4	43
	1.5	62	5.2	38
	1.1	55	4.6	45
	1.2	72	5.0	28
	1.3	72	5.6	28
	1.3	66	4.3	34
	1.6	58	4.9	42
	1.6	76	6.0	24
	1.0	78	3.5	22
	1.4	67	5.3	33
	1.6	63	5.1	37
	1.5	72	5.7	28
	1.4	73	5.5	27
700	0.6	81	2.9	19
	1.0	100	-	-
	0.6	82	3.1	18
	1.6	76	6.0	24
	0.5	75	2.7	25
	0.5	81	3.0	19
	0.4	82	2.7	19
	0.5	86	2.8	14
	0.3	71	1.9	29
	0.7	84	3.2	16
	0.4	80	2.9	20
	0.6	84	3.4	16
	0.5	63	2.5	37
	0.5	84	2.9	16
	0.6	82	3.4	18
	0.6	82	3.1	18
	0.5	84	3.0	16

[1] The error in lifetimes values is <10%.
